# Hunter-Gatherer Social Networks and Reproductive Success

**DOI:** 10.1038/s41598-017-01310-5

**Published:** 2017-04-25

**Authors:** Abigail E. Page, Nikhil Chaudhary, Sylvain Viguier, Mark Dyble, James Thompson, Daniel Smith, Gul. D. Salali, Ruth Mace, Andrea Bamberg Migliano

**Affiliations:** 1 Department of Anthropology, University College London, 14 Taviton Street, London, WC1H 0BW UK; 2 Institute for Advanced Study in Toulouse, 21 Allée de Brienne, 31015 Toulouse Cedex 6, France

## Abstract

Individuals’ centrality in their social network (who they and their social ties are connected to) has been associated with fertility, longevity, disease and information transmission in a range of taxa. Here, we present the first exploration in humans of the relationship between reproductive success and different measures of network centrality of 39 Agta and 38 BaYaka mothers. We collected three-meter contact (‘proximity’) networks and reproductive histories to test the prediction that individual centrality is positively associated with reproductive fitness (number of living offspring). Rather than direct social ties influencing reproductive success, mothers with greater indirect centrality (i.e. centrality determined by second and third degree ties) produced significantly more living offspring. However, indirect centrality is also correlated with sickness in the Agta, suggesting a trade-off. In complex social species, the optimisation of individuals’ network position has important ramifications for fitness, potentially due to easy access to different parts of the network, facilitating cooperation and social influence in unpredictable ecologies.

## Introduction

Direct social bonds (i.e. the relationship between A and B) are frequently associated with positive fitness outcomes including increased longevity, offspring survival and fertility in a wide range of animals including primates^1–3^, marine mammals^4–6^, insects^7^ and feral horses^8^. In humans, our friendships and social interactions are positively linked with increased longevity^9^, happiness^10^ and mental health^11^. Furthermore, there is a wealth of literature within human behavioural ecology denoting the importance of kin, particularly same-sex kin and grandmothers in terms of accessing cooperative breeding networks^12^, reducing maternal energetic expenditure^13^, increasing child survivorship and wellbeing^14–16^ and/or maternal fertility^17^. While these associations between social bonds and various measures of fitness are not consistently found^2^, it seems that among many gregarious species, who interacts with whom has important implications for various measures of wellbeing and fitness. Consequently, we might expect the optimisation of individual’s social network position to play an important role in reproductive success^7^.

**Figure 1:**
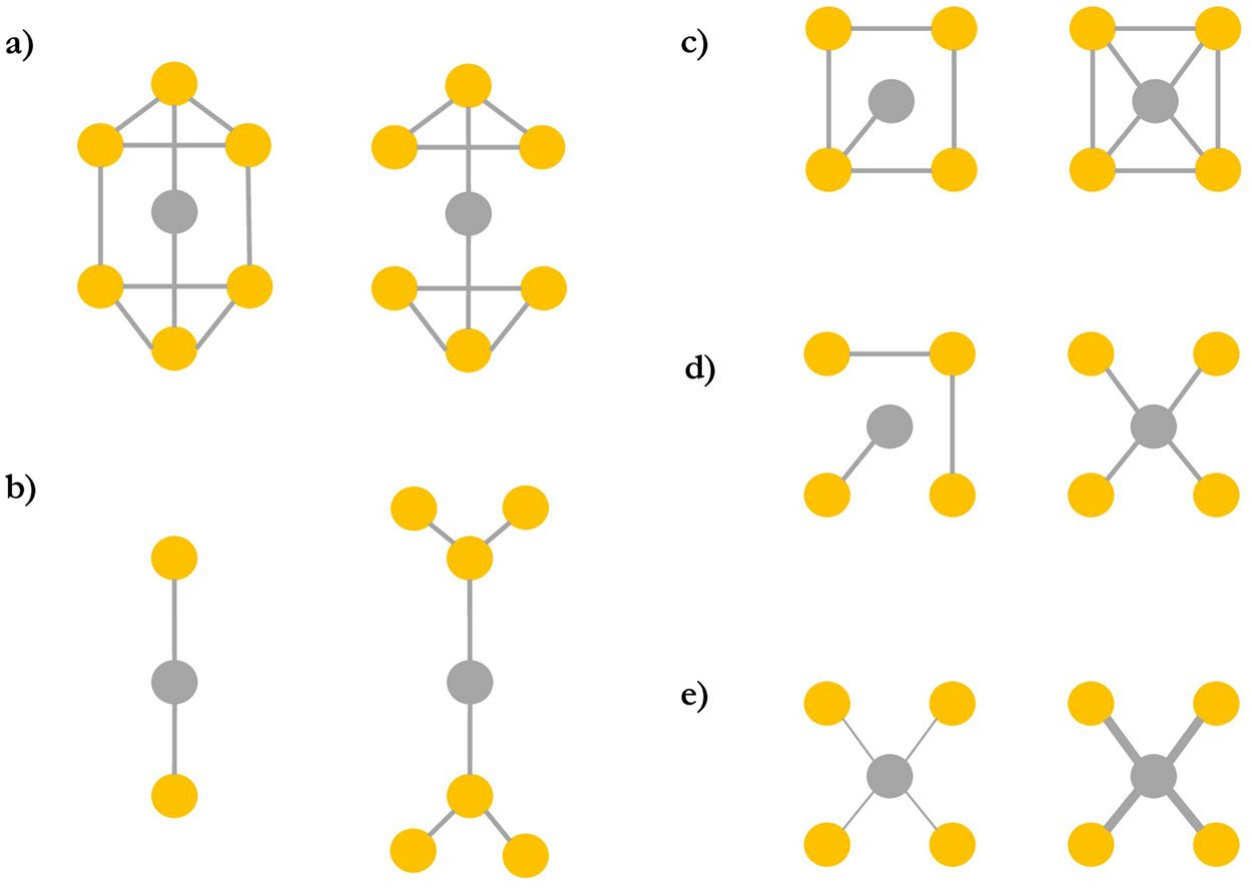
Visualisation of five measures of network centrality for (**a**) betweenness, (**b**) eigenvector centrality, (**c**) closeness, (**d**) degree and (**e**) strength. In each image the focal node is shaded grey and all other nodes yellow. The thickness of the tie represents the ‘strength’ of the relationship. In each measure of centrality, the image on the left represents low centrality, the right high. For instance, in (d) the figure to the left reveals that the focal ego is only tied to one other individual, while in contrast in the right figure the focal ego is connected to four nodes, thus representative of higher degree. Figures (d,e) are direct measures of centrality, the others are indirect. Adapted from ref. 19.

There are multiple measures of network position^18^, or ‘centrality’, some reflecting direct social ties (such as degree and strength) and others, indirect social ties, which extend to many more degrees of separation than the direct relationship between A and B^19^. *Degree* (Fig. 1) is the total number of an individual’s dyadic ties. An individual (in network terminology a ‘node’) with more social ties may experience more prestige^20^, increased cooperation or social tolerance which frequently translates into fitness gains^3, 21, 22^. An individual’s *strength* is the sum of all the tie weights (i.e. how often individuals interact), thus differentiates between strong and weak ties^23^. Thus far the literature has predominately explored the relative importance of strong or weak direct social ties and their relationship with social integration and bonding, resulting in positive fitness effects^8, 24, 25^: some have argued that a few strong social ties are a better predictor of fitness than many weak ties^26, 27^ while others point to the importance of broad social networks comprised of weak ties^28^.

The importance of such dyadic ties has been extensively studied within sociology and public health to understand the influence of social networks and social support on human behaviour, health and wellbeing. Social networks are understood to influence health and wellbeing via a multitude of pathways, from the provision of social support, gaining of social influence or more directly, via pathogen exposure or access to resources^29^. For instance, an insufficient social network, when experienced chronically, with too few dyadic ties has been associated with increasing stress levels, which activate physiological systems increasing the risk of a range of physical and psychiatric disorders^30^. The consequences of having few social contacts can be severe, with mortality risk significantly increasing in American adults reporting few social and community ties^31^. Furthermore, in a follow-up study of African American patients undergoing cardiac rehabilitation those with more social ties reported significantly increased coping efficacy and improved health behaviours^32^. Moving into the anthropological literature, it is evident that human reproduction is reliant on cooperative social networks^15^, as childcare from particular kin is associated with increased child survival^15, 33^, wellbeing^34^ and maternal fertility^17, 35, 36^. Thus, having many social bonds or strong bonds may be an important predictor of reproductive assistance, thus correlating with fitness in terms of increased child survival or maternal fertility.

Social networks are, however, more complex than direct social ties. For instance, who your friends are connected to and the degree of network clustering are impossible to examine by only exploring dyadic relationships^19^. Yet, these features play a central role in the transmission of information^6, 37^ and disease^38, 39^. Social network analysis examines how the interactions between individuals creates a structure which impacts the functionality of a system^20, 40^. Variation in individuals’ indirect centrality in the network results in differential access to any ‘currency’ (e.g. information, influence, disease, calories and resources) moved through the network^20, 40^. Thus, individuals with greater indirect centrality may benefit from increased and/or quicker access to this ‘currency’ and in turn achieve higher fitness^3, 41^.

Numerous measures of indirect centrality have been established, each of which quantify different attributes of an individual’s position within a network^18^. Here we explore eigenvector centrality, betweenness and closeness. *Eigenvector centrality* (EC) takes into account both the number and centrality of a node’s ties^42^. Nodes connected to other well-connected nodes have a higher EC centrality, as do nodes with many neighbours^19, 20^. Therefore, individuals with higher EC may have higher social status, or at least are associated with higher status individuals. Consequentially, EC has been positively correlated with infant survival in rhesus macaques (*Macaca mulatta* ^43^).

*Betweenness* is proportional to the number of geodesic (shortest) paths a node lies on between any other two nodes^20^. Thus, an individual with high betweenness can be considered a ‘broker’ in the network as they have a large influence on the flow of resources^19, 23, 42^. A second measure of indirect centrality is *closeness*, which is the inverse sum of the geodesic paths between ego and all other nodes^18^. Closeness represents the speed or efficiency (i.e. low distance) by which a focal node can reach all other nodes in the network^44^. Betweenness and closeness are highly correlated as they both measure node independence (i.e. high closeness is when the focal node does not have to travel through many other nodes to reach any given point in the network), which may be important for individual access to social support and influence^45^. As a result both have been associated with positive fitness outcomes in non-human taxa^1, 46^.

In societies without material wealth, such as extant human foraging populations, the importance of social networks is often highlighted as a means of buffering individuals from nutritional shortfalls in unpredictable environments^47–49^. Without wealth and/or food storage, foragers rely on cooperation to meet both short- and long-term calorific scarcity. For instance, among the Ache foragers of Paraguay an absence of food sharing resulted in the average household having less than 1000 cal per member on 27% of days. However, with food sharing this shortage is limited to only 3% of days^50^. Thus, social networks can be considered a form of insurance to mitigate resource deficits^51^. Foragers face resource shortfalls due to three factors: daily hunting and foraging success; illness and disability and cumulative dependency load^52^. For instance, in Headland’s^53^ Agta sample men were only successful on 21% of foraging trips, while this figure is as low as 3.4 for big game hunting in the Hadza^54^. Therefore, cooperative networks, which facilitate food sharing are essential to reduce the risk of daily shortfalls^55^. However, shortfalls also occur due to sickness and disease; individuals who are more cooperative, with larger cooperative networks are able to receive essential nutrients when they are unable to produce, buffering them from the negative consequences of failure to produce food over a few days or even a month^48, 56^.

Due to the importance of cooperation in small-scale societies social network structures have been demonstrated to affect the context in which individuals interact, and thus cooperate: allowing the assortment of cooperative individuals and the avoidance of defectors^57–59^. While direct social ties may be important for social integration^8^, indirect ties are more greatly influenced by network dynamics. Network dynamics form feedback loops, with individual behaviours influencing network structures, which in turn may facilitate cooperation, resulting in direct fitness consequences^58^. Therefore, we posit that the indirect structure of the network has important social implications; ‘well-placed’ individuals (those with higher indirect centrality) are better manipulators of their social network, gaining improved access to food, resources or political influence, directly influencing their reproductive success. However, these same network characteristics may also facilitate the transmission of negative currencies, such as disease^45, 60^ demonstrating the cost of social bonds.

We hypothesise that centrality in the network, particularly indirect centrality, is an important strategy to maximise and gain quicker access to key currencies that flow through the network which are essential for survival and reproduction in the unpredictable foraging context. While direct ties may be important, here we expect indirect ties to be more so as they directly influence the structure of cooperation and social interactions, which are key for hunter-gatherers^48^. As a result, we develop three independent predictions: 1) a positive association between indirect centrality and reproductive success; 2) a positive association between direct centrality and reproductive success; 3) network characteristics that are good for the flow of resources, may also facilitate the transmission of negative currencies, such as disease. Thus, measures of centrality may be associated with sickness.

Here, we explored maternal social network centrality using wireless sensing technology (motes^37^) and reproductive success among two foraging populations – the Agta from the Philippines (200 individuals, 7210 dyadic interactions) and BaYaka from the Congo (132 individuals, 3397 dyadic interactions). Motes record all dyadic interactions within a radius of approximately three meters at two-minute intervals for 15 hours a day (05:00–20:00) over the course of one week, producing high-resolution proximity networks mapping the totality of close-range interactions. From these networks, we created five common measures of centrality, which were explored as predictors of reproductive success (measured as number of currently living offspring).

For 39 Agta and 38 BaYaka mothers we found that network closeness and betweenness are positively correlated with number of living offspring in both populations. Although not explored among the BaYaka, among the Agta this network position appears associated with a significant cost, as more central mothers reported increased instances of sickness. We argue that in gregarious species the optimisation of social network position has important implications for individuals’ fitness, due to the importance of sociality and cooperation for reproduction and survival.

## Results

**Figure 2:**
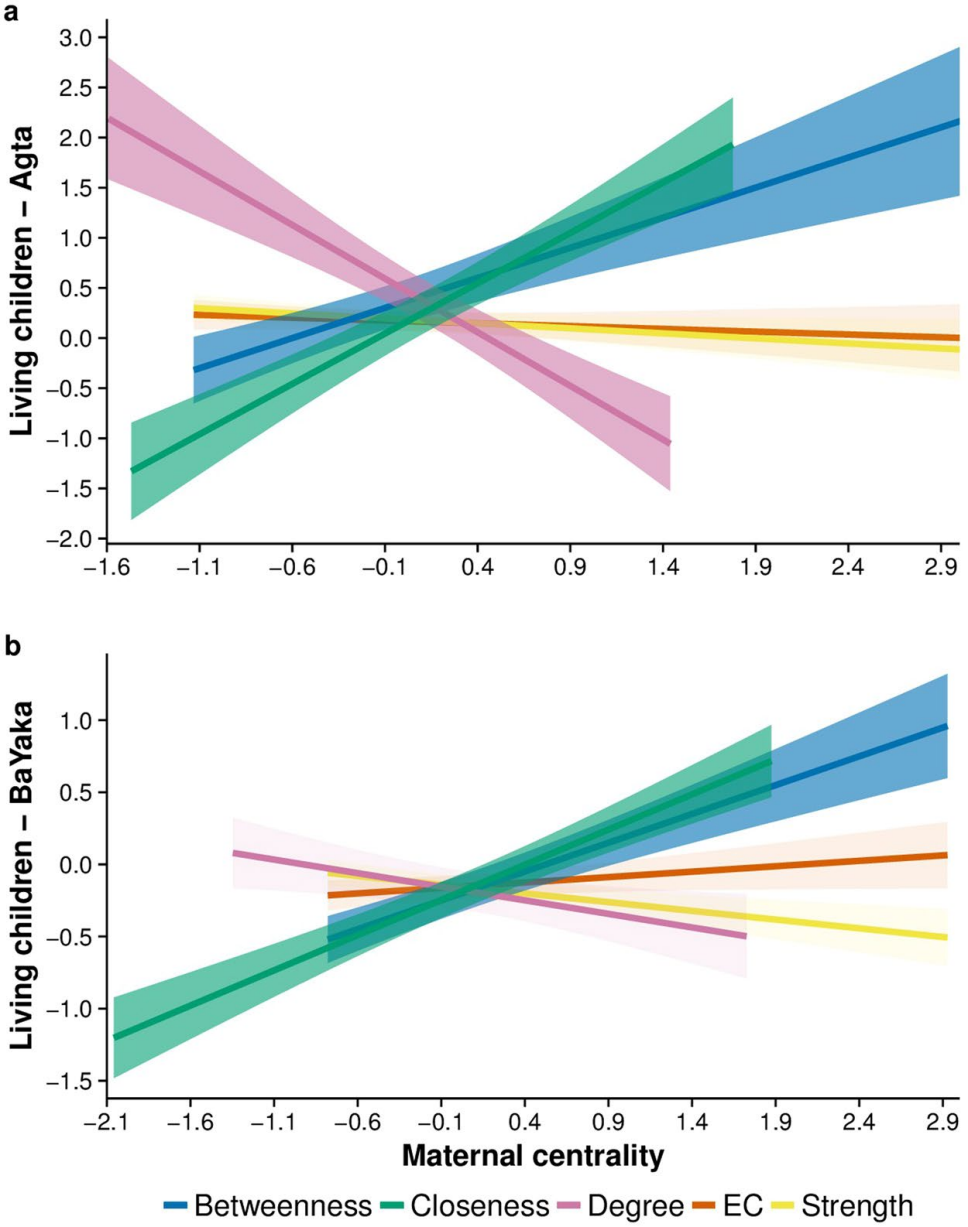
Age-controlled number of living offspring and five different measures of maternal centrality for (**a**) Agta (*n* = 39) and (**b**) the BaYaka (*n* = 38). Darker shaded areas represent significant results at *p* < 0.05. Shaded areas represent 95% confidence intervals.

Descriptive results are presented in Table 1. Indirect centrality as measured by betweenness and closeness centrality was positively associated with the number of living offspring in both the Agta and BaYaka (Table 2, Fig. 2). For the Agta, network centrality significantly interacted with age, demonstrating that the effects of betweenness and closeness on the number of living offspring progressively grew with age. In the BaYaka no such relationship with age was apparent. As revealed by Fig. 2, the relationship that betweenness and closeness hold with number of living offspring is extremely similar in the two populations. Accordingly, in a regression these two measures of centrality are positively correlated (Agta: B = 0.62, *p* < 0.001, adjusted R^2^ = 0.57; Congo: B = 0.78, *p* < 0.001, adjusted R^2^ = 0.63; for correlations between all centrality measures see Fig. S3).

**Table 1:** Descriptive statistics for the sample for Agta mothers (*n* = 39) and BaYaka mothers (*n* = 38).

Variable	Agta				BaYaka			
	Min.	Mean	Max	SD	Min.	Mean	Max	SD
Maternal Age	17.00	36.29	75.00	15.94	18.00	42.95	53.70	17.35
Betweenness	−1.13	−0.06	3.05	0.93	−0.78	0.09	2.93	1.07
Degree	−1.59	0.11	1.44	0.67	−1.35	0.01	1.73	0.89
Strength	−1.61	0.20	1.78	0.78	−1.08	−0.16	1.62	0.62
EC	−1.33	0.18	1.77	0.86	−0.83	−0.15	2.00	0.71
Closeness	−1.47	0.19	0.19	0.79	−2.06	0.05	1.87	1.04
Living offspring	−4.90	0.33	4.42	2.00	−2.69	−0.18	2.26	1.19
Cases of sickness	0.00	0.81	2.00	0.71	—	—	—	—

All network centrality measures are z-scores to standardise the results per camp. Living offspring are residuals from an analysis between age and living offspring, 0 representing the average fertility of the age group.

**Table 2:** Linear regression results for the relationship between five measures of centrality and age-controlled residuals for living offspring in the Agta and BaYaka.

	Agta (n = 39)				BaYaka (n = 38)			
	β	*p*	95% CI	Adjusted R2	β	*p*	95% CI	Adjusted R2
Degree	−1.5	0.019	−2.74, −0.26	0.23	−0.47	0.26	−1.31, 0.36	0.001
Degree*age	−2.577	0.053	−5.18, 0.03		—	—	—	—
Strength	−1.068	0.11	−2.39, 0.26	0.14	−0.395	0.331	−1.21, 0.42	0.019
Betweenness	2.445	<0.001	1.25, 3.64	0.46	0.872	0.029	0.1, 1.65	0.095
Between*age	6.025	<0.001	3.19, 8.87		—	—	—	—
EC	−1.07	0.103	−2.37, 023	0.14	−0.124	0.764	−0.95, 0.71	0.047
Closeness	1.674	0.007	0.49, 2.85	0.31	0.962	0.015	0.20, 1.73	0.125
Close*age	3.613	0.011	0.89, 6.34		—	—	—	—

Age is mean centred at 36 years in the Agta and 41.7 years in the BaYaka. Models control for camp membership and all betas are standardised.

Contra predictions, degree centrality was negatively correlated with reproductive success, a relationship again dependent on age in the Agta. As different measures of centrality capture aspects of the same network dynamics^61^ degree was modelled with betweenness and closeness to explore which were the strongest predictors of reproductive success (while ensuring that multicollinearity was at acceptable levels, discussed further in the methodological section and variance inflation factors are presented in the SI^62^). Among the Agta, betweenness (β = 2.48, *p* < 0.001, 95% CI [1.15, 3.81]) and closeness (β = 1.55, *p* = 0.015, 95% CI [0.33, 2.77]) retained their positive association with number of living offspring, while degree was no longer a significant predictor (β = −0.59, *p* = 0.36, 95% CI [−1.88, 0.71]). Among the BaYaka closeness remained a significant predictor (β = 0.93, *p* = 0.034, 95% CI [0.08, 1.78]). However, the inclusion of degree (which was non-significant: β = −0.08, *p* = 0.85, 95% CI [−0.99, 0.82]) resulted in betweenness becoming marginal (β = 0.83, *p* = 0.063, 95% CI [−0.05, 1.72]), suggesting closeness is the stronger predictor of living offspring. Full model results are presented in the SI.

Mothers with the highest betweenness and closeness in the Agta reported significantly more instances of sickness. However, this relationship appeared significantly mediated by number of living offspring for betweenness. Number of dependents significantly predicted cases of reported sickness (β = 0.73, *p* = 0.006, 95% CI [0.24, 1.23]), simultaneously removing the significance of betweenness (β = 0.27, *p* = 0.2, 95% CI [−0.16, 0.72]). Nonetheless, the relationship between closeness and instances of sickness remains significant even with the inclusion of number of living dependents (β = 0.3, *p* = 0.045, 95% CI [0.01, 0.6]), suggesting that individuals with higher closeness experience more cases of sickness independent of family size. No other measure of maternal centrality significantly predicted cases of sickness, nor did any measures significantly interact with age (see SI).

## Discussion

By exploring maternal centrality in high-resolution proximity networks, we have provided the first evidence, to our knowledge, for fitness implications of network centrality in hunter-gatherers. Specifically, we find that betweenness and closeness positively predicted number of living offspring in both the Agta and BaYaka. However, these network positions also appeared to be associated with detrimental health outcomes as individuals most ‘closely’ connected to all other nodes experienced increased instances of sickness.

Both betweenness and closeness share properties of independence and efficiency: optimizing the speed and ease at which any individual can reach throughout the network, reducing the cost of connectivity and perhaps promoting social coordination and access to cooperation^63^. These features may be particularly important in cooperative systems, such as found in human foragers, suggesting possible mediating links between centrality and fertility. If, as argued, forgers buffer risk and stochasticity in unpredictable environments with extensive cooperation^34, 47, 64^ then the structure of an individuals’ cooperative social networks may impact how effectively they can surmount these ecological challenges^51^. However, the relationship between centrality and reproductive success may be ecologically variable: behaviour and social networks are all highly flexible, thus centrality may permit plasticity in behavioural strategies according to need^25^.

Comparable results have been found among free-ranging chimpanzees (*Pan troglodytes schweinfurthii*), where male betweenness in coalition membership correlated with increased rank and probability of siring offspring^1^. Males who ‘bridged’ otherwise unconnected coalitions appeared to maximise their connectivity, indicating that avoiding coalition formation against males with shared partners had positive fitness consequences. Thus, the structure of cooperative networks may be an important mediator between centrality and fitness. Closeness has also been associated with positive fitness outcomes^46^, as have other indirect network measures which capture similar structural properties (i.e. information centrality and reach^45, 46, 65^), indicating the importance of indirect social ties in a range of taxa.

This study, however, does not test why indirect centrality may be correlated with reproductive success. Thus, inferences about cooperation are limited. This study does test an *a priori* hypothesis that in social species, complex and indirect social relationships influence individuals’ reproductive success. This hypothesis has been supported. However, many indirect measures of centrality may be by-products of other traits correlated with fitness. For instance, higher quality mothers may have higher centrality due to increased social status and prestige, which have well-known associations with fertility^22, 64, 66^. Nonetheless, the relationships between social status, cooperation and fitness outcomes can be interconnected. For instance, among the Tsimane hunter-horticulturalists, politically influential men demonstrated significantly lower cortisol levels, due to increased social support networks^67^. This indicates the influential role of social networks and social status on different fitness outcomes.

Centrality does not, however, come without its costs as Agta mothers with greater betweenness and closeness appear to suffer from more bouts of sickness. This finding is in line with much of the literature on disease transmission which finds that ‘brokers’ in the network are more likely to host a pathogen^38, 45, 60, 68^. As individuals with high betweenness are those who lie on central ties, it follows that much of the disease transmission flows through them^60^. However, the sickest mothers were also those with more children, suggesting a trade-off between fertility and somatic maintenance^69^ or children’s role as ‘super-spreaders’ of disease^39, 70^. Nonetheless, closeness centrality is independently correlated with self-reported sickness in other species. For instance, closeness has been found to be important in transmission of *Mycobacterium bovis* (TB) in brushtail possums (*Trichosurus vulpecula*) given individuals rapid access to all other network nodes^45^. Thus, while central individuals may receive higher fitness overall, they do face increased disease burdens in the process. Consequently, individuals must trade-off between rapid access to ‘relational wealth’ versus a rapid transmission of pathogens, particularly in high morbidity, mortality environments such as those that the Agta^71^ and central African Pygmies reside in ref. 72.

The key limitation of this study is its correlational nature: further research needs to be conducted into the processes underlying these associations to understand functionality. We hypothesise that in hunter-gatherers cooperative relationships are essential for reproductive success. If that is the case, future research should examine the mediating role of cooperative behaviours, exploring how social networks vary over time according to reproductive stages, thus better separating out different causal pathways. For instance, do mothers with many children seek centrality to ensure cooperative childcare ? This will also shed further light onto the significant age interaction between centrality and living offspring among the Agta. Several lines of evidence suggest that centrality can be maintained over the life-course or even between generations^43, 73^. Furthermore, early life centrality is associated with fitness outcomes in later life in long-tailed manakins (*Chiroxiphia linearis* ^65^), bottlenose dolphins (*Tursiops truncatus* ^6^), while in humans having larger networks of friends was protective against mortality in a ten-year follow up period^74^. Therefore, if the fitness effects of social networks are a product of lifetime centrality then their effects may accumulate over the life course. Why this interaction is not significant among the BaYaka is unclear, however the BaYaka have a significantly older population distribution, perhaps obscuring these effects given our small sample sizes.

Another limitation is the duration of this study. A one-week snapshot may not be reflective of a typical week for all the individuals in the sample. However, this is the first time such wireless sensing technologies have been used with this purpose in foraging populations, capturing a significantly larger and denser sample for social network analysis than previously possible. In the childcare observational studies, for instance, samples sizes are often limited to 15 to 25 children^75, 76^ who are observed for a total of 9 hours^77, 78^. Therefore, by utilising the motes we produce significantly larger and longer observational samples. As social networks are the product of behavioural strategies we should expect them to be flexible and reactive to challenges in the ecology. Therefore, it is necessary to consider the fact that the timescale of our response and predictor variables differ; data on network centrality are snapshot measures, whereas measures of reproductive success reflect the entirety of an individual’s reproductive career. Continued research into the dynamic and changing nature of social networks is essential to explore these questions further.

We have shown that individuals’ network centrality is associated with fitness outcomes among two foraging populations. This reveals how indirect ties have important relationships with fitness in complex social systems. Given the variable and unpredictable hunter-gatherer environment the ability to manipulate one’s social network may offer an important insight into the evolution of sociality and cooperation^79^. These findings hint at the evolutionary importance of social intelligence in primates^80, 81^: species dependent on coordination, knowledge transfer and social learning for cooperation and other fitness promoting traits, would benefit from ease of access throughout the network promoted by centrality^63^. Thus, awareness of who is friends with whom may have important fitness implications in social primates, and as such dynamics take considerable social intelligence, this indicates possible selective pressures for brain expansion in primates^82^. These results are suggestive of the evolutionary importance of encephalisation in facilitating management of complex and diverse social networks since an individual’s centrality depends not only on their direct ties but also indirect ties throughout the population^7^. Further research using social network analysis to explore these indirect properties’ influence on human fitness is essential, as they may play a major role in our social and behavioural evolution.

## Methods

### Study Populations

#### The Agta

Data collection occurred over two field seasons from April to June 2013 and February to October 2014. There are around 1,000 Palanan Agta living in Isabela Province, located in the northeast of Luzon, in the Philippines. The Agta reside in the Northern Sierra Madre Natural Park (NSMNP), a protected area that consists of a mountainous tropical rainforest and includes the coastal beaches, coral reefs and the marine eco-system of the Pacific Ocean. Similar to many immediate-return hunter-gatherer societies worldwide the Agta follow a bilateral descent and residence system, which maintains a large and flexible kinship network^83–86^. Having such a large kinship base allows easy access to collectively held land as family groups are mobile, and often move between different camps on a regular basis^85^. Peterson^86^ notes that factors, such as food availability and personal relations meant that nuclear families move between three to five camps within a delimited locale. In our own data, we found that, on average, households move once every 10 days. The ability to be mobile is essential in facilitating cooperation^87^, and while there is variability in the types of cooperation the Agta are highly cooperative in terms food sharing between individuals, households and the wider camp as well as engaging in cooperative hunting^55, 88^.

The Agta rely heavily on foraging modes of subsistence (76.5%) versus non-foraging activities (23.5%). Riverine and marine spearfishing provides the primary source of animal protein, supplemented by inter-tidal foraging, hunting and the gathering of wild foods as well as low-intensity cultivation^85^. As a result, on average 19.6% of food is produced from cultivation while the remaining 80.4% is produced by foraging activities (fishing, hunting and gathering). The Agta have long resided with neighbouring farming populations, trading meat for rice and, historically, tubers^86^.

#### The Mbendjele BaYaka

The Mbendjele BaYaka are a subgroup of the BaYaka and reside in an area spanning northern Republic of Congo and southern Central African Republic. The three camps described in this paper are situated in the Sangha and Likouala regions of the Congo rainforest. Among the Mbendjele, hunting in the forest is the primary source of animal protein, men also climb to collect calorie rich honey. Women make significant contributions to the diet by gathering plants, digging tubers and fishing. The Mbendjele also trade forest products for manioc, alcohol and cigarettes with neighbouring farmer groups.

Similar to many hunter-gatherer populations, including the Agta, the Mbendjele are highly mobile and live in camps of fluid membership containing a large proportion of unrelated individuals^83^. Their social organisation is described as being ‘fiercely egalitarian’^89^, and this egalitarianism extends across ages and sex. Food sharing is also extremely prevalent in Mbendjele camps owing to highly variable foraging returns, necessitating significant food transfers and cooperation to buffer nutritional shortfalls – on average 36.8% of a households production is shared with non-household members^55^. In fact, in a meta-analysis of human and non-human primate reciprocal food sharing, reciprocal transfers were found to be more prominent in a BaYaka group (the Aka) than any other included in the study^90^.

### Data collection

We stayed approximately 14 days in six Agta camps and three BaYaka camps to collect data on both reproductive histories and social interactions.

#### Reproductive success

To establish a measure of reproductive success we conducted reproductive histories with 39 Agta and 38 BaYaka mothers. We enquired about all currently living offspring (of all ages), producing a proxy of reproductive success as it captures both fertility and early life survival.

#### Motes

Social networks were captured using ‘motes’ (wireless sensing devices) which communicate with one another and store all communications within a specified distance^37^. The device we utilised was the UCMote Mini (Unicomp Ltd, Standford, USA). Each device sends a message that contained its unique ID, a time stamp and the signal strength at a programmed interval (every two minutes). This message is picked up and stored by any other mote within a three-meter radius around the emitting mote. At the end of the experiment these data are downloaded for analysis. Three metres cut-off for proximate interactions as it is a common threshold used in interaction studies^13^ to denote dyadic exchanges. Therefore, this threshold captures close proximity which is necessary for important interactions, such as childcare, playing, hunting, foraging, cultural exchange (i.e. showing, learning and sharing) as well as disease transmission^91^.

The motes were sealed into wristbands and belts (depending on size and preference, Fig S1). The motes experiment was undertaken in one camp at a time. Each mote was labelled with a unique number and identified with coloured string to ensure swaps did not occur. All individuals within a camp wore the motes from a period ranging from five to nine days depending on the camp. While the motes were worn throughout the night, data was only selected from between 5:00 and 20:00. This was to avoid long hours of simply recording who slept in the same shelter. If individuals arrived at a camp during the experiment they were promptly given a mote, and entry time was recorded. Similarly, if an individual left a camp at any time before the end of the experiment, the time they returned the mote was recorded. To ensure swaps did not occur individuals were regularly asked to check they were wearing the correct armband. All mote numbers were also checked when they were being handed back to ensure we always knew who had worn each mote. Any swaps were recorded during the experiment and adjusted in the final data processing. Validation of the motes can be found in the SI.

### Medical survey

Among the Agta we conducted a medical survey based on sickness symptoms over the last two weeks, focusing on gastro-intestinal disease, influenza and fever, respiratory tract infections and intestinal parasites. After data collection with a qualified health care assistant the completed questionnaire was handed back to the field doctor for diagnosis. The total number of medical diagnoses for each individual was calculated, which varied between zero to two instances of sickness. To control for wealth effects, we also recorded key household belongings present in each house during interviews (further information in the SI).

### Ethics

This research and fieldwork was approved by UCL Ethics Committee (UCL Ethics code 3086/003) and carried out with permission from local government and tribal leaders in Palanan and the Congo. All methods were performed in accordance with the UCL ethics guidance and regulations. Informed consent was obtained from all participants, and parents signed the informed consents for their children (after group and individual consultation and explanation of the research objectives in the indigenous language). All diagnosed medical conditions were treated in association with the local field hospital. A small compensation (usually a thermal bottle or cooking utensils) was given to each participant when the mote was returned at the end of the experiment.

### Analysis

All data preparation, social network analysis and statistical analysis was conducted in R version 3.1.2^92^ using the *igraph* package for social network analysis. The raw frequency of interaction data was transformed from a dyadic matrix to a social network graph for the computation of centrality measures. This raw data was adjusted for time present in camp of both individuals in the dyad, to control for individuals arriving to camp during the experiment or leaving the experiment early. The social network only comprised of individuals aged 12 years or older. This threshold was applied as after the age of eleven, hunter-gatherer children conduct significantly more caring and economic activities. They also require less care and provisioning themselves^93^. All network measures were standardized by camp, thus represent whether or not a centrality score was high relative to the camp average^5, 94^. Given that most camps were small, almost all individuals had some level of interaction with each other. Therefore, degree centrality was computed from the ties which were greater than 1% of recorded weighted interactions.

#### Measuring the effects of mothers’ social network position on living offspring

We used number of living offspring as it captured both fertility and child survivorship and is, therefore, our best measure of reproductive success. Due to differences in data collection between the two fieldsites we were unable to use a more robust measure of fitness (survivorship to age 16) as we did in previous work^71^, because the BaYaka dataset does not include mortality data. Therefore, number of currently living offspring was used in both populations. In order to control for the relationship between age and reproductive success, we removed the effect of age on fertility by producing age-specific fertility residuals from non-linear models. Generalised linear models were run with the dependent variable of living offspring and predictors of age and the square of age to capture the quadratic nature of the fertility distribution. All models were run with a Poisson distribution due to the discrete nature of the data. These residuals had no significant relationship with age and its quadratic term (*p* = 1.0 in all cases) after this transformation. The age-specific residuals produced from the raw living offspring data allowed us to explore how high or low an individuals’ reproductive success is given their age. Here, a residual of 0 represents a woman with the average number of living offspring for her given age, negative values represent below average number of living offspring for one’s age, while positive residuals are above average.

Living offspring residuals formed the dependent variable in multivariate linear regressions with the five measures of network centrality as independent variables (degree, strength, EC, betweenness and closeness). These models contained a discrete variable of camp residence to capture any camp specific effects and all models met normality assumptions demonstrating the suitability of linear regressions (Table S1). Exploration of the Agta data revealed that the relationship between network centrality and number of living offspring demonstrated a significant interaction with age. As a result, in both datasets two models were run, one containing the interaction effect between centrality and age, and one without. In all cases if the interaction was insignificant at *p * >0.05 then the interaction was removed from the model and the non-interaction model is reported (non-significant models reported in the SI). Given the interaction term, age was mean centred (36 years in the Agta, and 41.6 in the BaYaka) to ease interpretation. When interactions are run the coefficients for the main effects become dependent on the interaction term, therefore it is necessarily to standardise the variables so that the main interaction effects can be interpreted in the same model^95, 96^. Therefore, all models are standardised over two standard deviations allowing for easy comparison of the different predictor effects. As the resulting coefficients are equal to the mean +1 standard deviations they are directly comparable to untransformed binary predictors^97^.

As many of the network centrality statistics co-vary, each of these terms were run in separate analyses initially^19, 61^. Multicollinearity occurs when independent predictors in a model are correlated resulting in biased parameter estimates, which become extremely sensitive to small modelling changes, making interpretation difficult. This occurs when variance inflation factors rise above 2.5^62^. Therefore, after the models were run separately we explored running degree, betweenness and closeness in the same model while ensuring that variance inflation factors remained under 2.5 (Tables S4 and S5). As a result, in these second models collinearity is not resulting in biased parameter estimates.

#### Measuring the effects of mother’s social network position on frequency of illness

The reported sickness models (Agta only) sought to explore the relationship between network centrality and sickness. In these models the dependent variable was number of instances of sickness in the past two weeks and the independent variables were the different measures of network centrality. These models controlled for maternal age, whether the camp was ‘settled’ (binary, 1 being settled representing a camp with permanent housing and a church and/or water pump), individual mobility (binary, 1 never witnessed to move camp during two year research period) and ‘household belongings’ (a continuous measure quantifying wealth) as these have known relationships with health and wellbeing^71^. Such controls were also originally included in the living fertility models, however as they had no influence on any model parameters they were removed to produce the most parsimonious model. Finally, number of children in the household was included in the model to control for the influence of high parity on maternal health. Descriptive statistics for all variables from both populations can be found in Table 1.

## Electronic supplementary material

Supplementary information
