# Supplementary material for: Hunter-Gatherer Social Networks and Reproductive Success
Source: Sci Rep. 2017 Apr 25;7:1153. doi: 10.1038/s41598-017-01310-5 (PMC5430806; doi:10.1038/s41598-017-01310-5)
Supplement: Supplementary file 1 — Supplementary information [file 41598_2017_1310_MOESM1_ESM.pdf]

Hunter-Gatherer Social Networks and Reproductive Success: Supplementary information

Authors: Abigail E. Page*, Nikhil Chaudhary, Sylvain Viguier, Mark Dyble, James Thompson, Daniel Smith, Gul. D. Salali, Ruth Mace and Andrea. Bamberg Migliano

Authors' affiliation: Department of Anthropology, University College London, 14 Taviton Street, London WC1H 0BW, UK.

Correspondence to: abigail.page.10@ucl.ac.uk

SI methods

Household belongings

To create an 'emic' based list, we first sought to establish the most important items from a sub-sample (n = 16) of households. We asked each household to name 10 of the most important belongings an Agta could own. Based on this we created a list of 14 household items that were mentioned the most frequently. This list was then shown to each household, asking whether they had these items and if they did, how many did they have. As some items were more important than others we weighted each item according to the number of times it appeared in the list. For instance, as most households owned cooking pots, a family without one would be considered quite 'poor' since these are an essential daily item. Thus, these items were weighted the highest. This system assumes that the 'most common' are the most valued, since it would be erroneous to compare cooking pots to spoons 1-to-1. However, it does undervalue rare, luxury items (such as radios or guns). The object, count and proportion can be found in Table S1. Overall, this method was thought to be more nuanced than taking the monetary value of items since this is unlikely to be directly reflective of the value the Agta place in the items.

Table S1: List of household objects and their weighting used in creation of household belonging variable.

Item	*n*	Proportion	Weight
Goggles	31	0.053	5
Blanket	37	0.063	6
Hunting bow	7	0.012	1
Cups	65	0.111	11
Air gun	5	0.009	1
Kettle	45	0.077	8
Knife	65	0.111	11
Mat	15	0.026	3
Net	12	0.020	2
Plates	93	0.158	16
Cooking pot	123	0.210	21
Radio	4	0.007	1
Spear gun	35	0.060	6
Spoon	50	0.085	9
Total	587	1.000	

Motes

A **B**

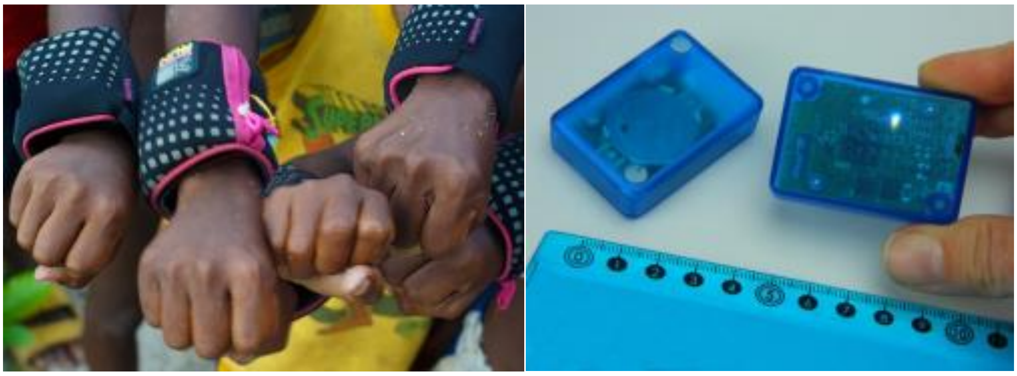

Figure S1: Mote utilisation in the field. (A) Motes switched on before packaging and (B) Agta children wearing their motes in armbands. Credit: Rodolph Schlaepfer and Sylvain Viguier

Comparison of motes data to observed proximity data

The innovative usage of remote sensing technologies to create high-density proximity networks required justification. Therefore, to establish whether or not the motes were, in fact, recording proximity at approximately three meters we compared this data to observational data from five toddlers (aged between two to five years) produced using focal sampling techniques (Meehan 2005; Meehan *et al*. 2013b; Fouts *et al*. 2005; Hewlett *et al*. 2000). In this technique a focal child is observed for 12 hours over several days to ensure a range of activities are captured. This 12-hour period is broken into three 4-hour intervals (6:00 – 10:00, 10:00 – 14:00 and 14:00 – 18:00) during which, the researcher records who is interacting with a child and what type of interaction this is every 20 seconds (observe for 20 seconds, record for 10 seconds) within a three meter radius. These 4-hour intervals were conducted on non-consecutive days to reduce any sampling bias (i.e. the father was out of camp for those two days). Due to the intensive nature of the data collection, 15-minute breaks are essential every hour, thus in total each child was observed for 9 hours. This produces 1,080 observational points per child over three days, compared to an average of 3,150 mote points over one week.

Table S2: Proportion of time toddlers spent with any given kin category for motes and focal observations. GP refers to grandparents. Non-kin are all individuals related less than r = 0.125, and other kin between 0.25 and 0.125. Categories that include multiple individuals (such as GP, siblings, other and non-kin) are summed across category, thus toddlers spend 23% of time with all non-kin, however, on average they spend only 2% of their time with any given non-kin individual. These proportions are not out of 1 since children can often be with more than one individual.

	Motes	Focals
**Mother**	0.34	0.37
**Father**	0.11	0.19
**GP**	0.06	0.02
**Siblings**	0.24	0.24
**Other kin**	0.07	0.08
**Non-kin**	0.23	0.24

To compare these two types of data, means were produced for the proportion of time five toddlers spent with specific kin categories. These differences are minimal, and the distribution of observations with specific kin types is not significantly altered between the two methods. For instance, the motes recorded that toddlers spent on average 34 $\pm$ 26% of time with mothers, 11 $\pm$ 5% of time with fathers, 24 $\pm$ 13% of time with siblings and 6 $\pm$ 6%, 7 $\pm$ 7% and 23 $\pm$ 13% for grandparents, other kin ($r \leq 0.25$ and ≥ 0.125) and non-kin ($r < 0.125$), respectively (Table S2). These same toddlers were observed to spend 37 $\pm$ 26% of time within three-meters of their mothers, 19 $\pm$ 19% with fathers, 24 $\pm$ 19 % with siblings and 2 $\pm$ 1%, 7 $\pm$ 8% and 24 $\pm$ 20% of their time with grandparents, other kin and non-kin, respectively (Figure S2). Therefore, the two types of data collection produce remarkably consistent and similar pictures of proximity at three meters. Overall, the consistency between the observational and motes data leads us to conclude motes have a high reliability and represent proximity at approximately three meters.

A

B

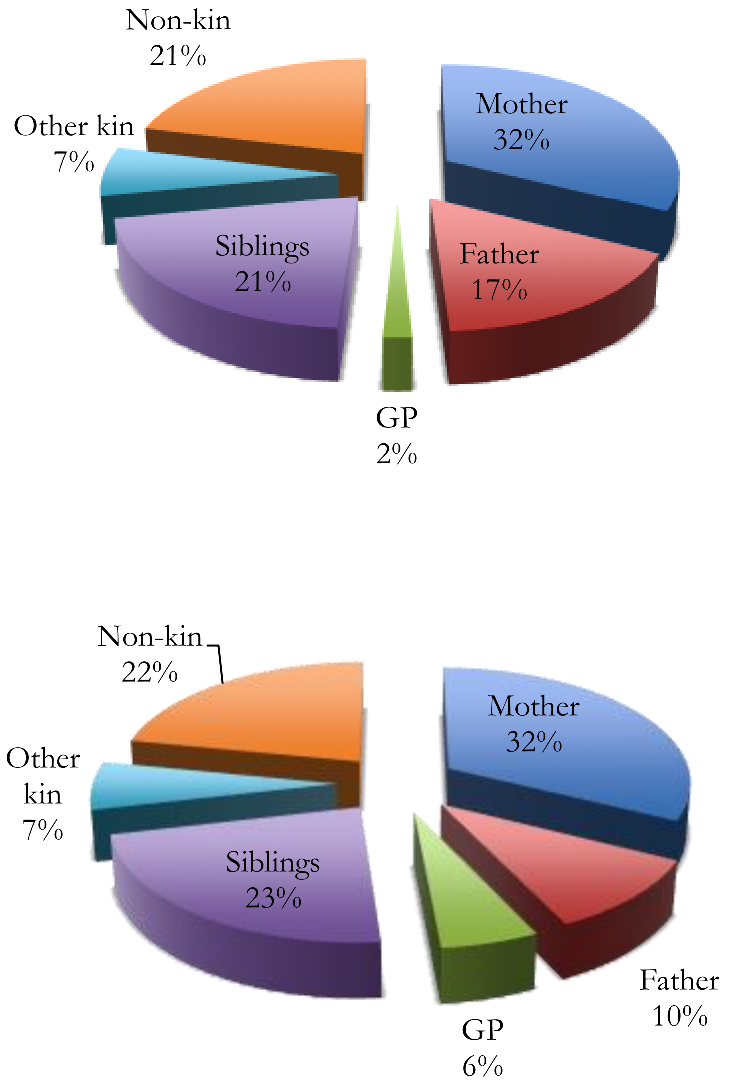

Figure S2: Proportion of time toddlers spent with different kin categories for data collected by (A) focal observations and (B) motes. GP represents grandparents.

SI Results

Model normality results

Table S3: Results from Shapiro-Wilk normality tests for each of the models reported. As no tests reach statistical significance ($p < 0.05$) this reveals that all models met the assumption in linear models that the residuals are normally distributed.

		Shapiro-Wilk Normality Test	
		W	*p*
BaYaka	Degree	0.963	0.249
	Strength	0.972	0.461
	Betweenness	0.956	0.154
	EC	0.980	0.727
	Closeness	0.954	0.126
Agta	Degree	0.969	0.365
	Strength	0.983	0.811
	Betweenness	0.971	0.426
	EC	0.985	0.885
	Closeness	0.984	0.839
Sickness	Degree	0.976	0.625
	Strength	0.953	0.144
	Betweenness	0.966	0.352
	EC	0.945	0.091
	Closeness	0.975	0.608

Variance inflation scores

Table S4: Betweenness VIFs when degree was also included in the model.

Betweenness VIFs			
**Variable**	**VIF**	**Df**	**VIF^(1/(2*Df))**
Betweenness z-score	1.523621	1	1.23435
Age centered	1.805471	1	1.343678
Degree z-score	1.164511	1	1.079125
Camp	1.161493	5	1.015083
Between*age	1.841236	1	1.356922

Table S5: Closeness VIFs when degree was also included in the model.

Closeness VIFs			
**Variable**	**VIF**	**Df**	**VIF^(1/(2*Df))**
Closeness z-score	1.254917	1	1.120231
Age centered	1.037709	1	1.01868
Degree z-score	1.300208	1	1.140266
Camp	1.315268	5	1.027783
Between*age	1.386373	1	1.177444

Correlations of centrality

As revealed by Fig. S3 betweenness and closeness are significantly positively correlated in both populations. Strength, degree and eigenvector centrality are strongly positively correlated. Therefore, measures of how many social ties individuals had, or how strong these ties are capture similar trends. Closeness was negatively correlated with degree, strength and EC (however not in the BaYaka data). While in the BaYaka betweenness is negatively correlated with degree this relationship is not significant in the Agta dataset, but reveals the same overall trends. As a result, betweenness and closeness represent similar network trends, in rough opposition to the trends presented by degree, strength and EC.

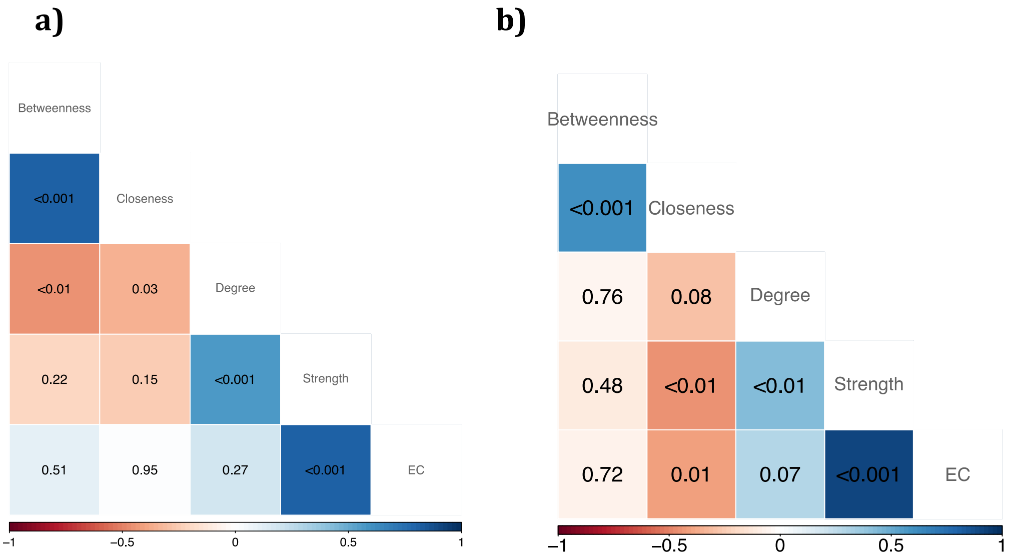

Figure S3: Correlation plot for the five different measures of network centrality for A) 38 BaYaka mothers and B) 39 Agta mothers. Darker shades represent stronger correlations, blue shades positive correlations, red shades negative correlations. Each correlation box contains the relevant dyadic p-value.

Full model outputs
All models were run with an interaction with age. If the term was not significant the non-interaction model was presented in the main text. Models are presented with relevant AIC value for model comparison.

Agta

EC:

	Interaction model				Non-interaction model			
	B	P	CI	CI	B	P	CI	CI
(Intercept)	2.544	0.070	-0.226	5.313	2.793	0.041	0.116	5.470
EC	-1.130	0.093	-2.460	0.199	-1.070	0.103	-2.371	0.230
Age	0.390	0.558	-0.957	1.737	-	-	-	-
Camp 1	-1.808	0.284	-5.196	1.580	-2.029	0.219	-5.326	1.269
Camp 2	-3.283	0.039	-6.390	-0.176	-3.593	0.020	-6.583	-0.604
Camp 3	-2.358	0.149	-5.606	0.891	-2.558	0.110	-5.725	0.608
Camp 4	-2.461	0.109	-5.504	0.582	-2.687	0.073	-5.643	0.269
Camp 5	-1.642	0.285	-4.721	1.438	-1.970	0.183	-4.921	0.981
EC*age	-1.318	0.326	-4.017	1.380	-	-	-	-
AIC	165.614				163.012			

Strength:

	Interaction model				Non-interaction model			
	B	P	CI	CI	B	P	CI	CI
(Intercept)	2.623	0.063	-0.157	5.402	2.906	0.035	0.214	5.598
Strength	-1.378	0.063	-2.838	0.082	-1.068	0.110	-2.393	0.256
Age	0.316	0.627	-1.002	1.634	-	-	-	-
Camp 1	-2.084	0.221	-5.491	1.322	-2.240	0.182	-5.586	1.105
Camp 2	-3.329	0.038	-6.451	-0.206	-3.720	0.017	-6.716	-0.724
Camp 3	-2.505	0.126	-5.755	0.745	-2.697	0.094	-5.881	0.488
Camp 4	-2.547	0.097	-5.581	0.487	-2.766	0.066	-5.727	0.195
Camp 5	-1.716	0.265	-4.805	1.373	-2.073	0.165	-5.048	0.902
Strength*age	-1.845	0.276	-5.245	1.554	-	-	-	-
AIC	165.493				163.138			

Degree:

	Interaction model				Non-interaction model			
	B	P	CI	CI	B	P	CI	CI
(Intercept)	1.465	0.272	-1.212	4.141	2.186	0.103	-0.470	4.842
Degree	-1.499	0.019	-2.735	-0.263	-1.297	0.044	-2.553	-0.040
Age	0.392	0.517	-0.831	1.615	-	-	-	-
Camp 1	-0.237	0.883	-3.501	3.028	-0.957	0.556	-4.238	2.324
Camp 2	-2.327	0.122	-5.309	0.656	-3.121	0.040	-6.083	-0.159
Camp 3	-1.616	0.289	-4.671	1.440	-2.129	0.172	-5.234	0.976
Camp 4	-1.523	0.296	-4.446	1.401	-2.127	0.151	-5.071	0.818
Camp 5	-0.232	0.874	-3.207	2.743	-1.188	0.410	-4.087	1.712
Degree*Age	-2.577	0.053	-5.184	0.029	-	-	-	-
AIC	160.181				161.241			

Betweenness:

	Interaction model				Non-interaction model			
	B	P	CI	CI	B	P	CI	CI
(Intercept)	3.069	0.007	0.922	5.215	2.946	0.032	0.277	5.615
Betweenness	2.445	0.000	1.247	3.643	1.151	0.077	-0.133	2.434
Age	1.715	0.007	0.506	2.923	-	-	-	-
Camp 1	-1.367	0.295	-3.986	1.252	-1.767	0.276	-5.017	1.482
Camp 2	-3.340	0.008	-5.731	-0.948	-3.718	0.016	-6.686	-0.751
Camp 3	-2.841	0.029	-5.378	-0.304	-2.729	0.088	-5.884	0.427
Camp 4	-3.131	0.012	-5.517	-0.744	-3.111	0.040	-6.076	-0.146
Camp 5	-1.998	0.095	-4.365	0.369	-2.088	0.158	-5.030	0.855
Between*age	6.026	0.000	3.185	8.867	-	-	-	-
AIC	147.078				162.425			

Betweenness and degree:

	Betweenness and degree model			
	B	P	CI	CI
(Intercept)	3.107	0.009	0.850	5.364
Betweenness	2.480	0.001	1.156	3.805
Age	1.750	0.012	0.414	3.086
Degree	0.089	0.889	-1.208	1.386
Camp 1	-1.409	0.301	-4.147	1.329
Camp 2	-3.366	0.009	-5.833	-0.899
Camp 3	-2.866	0.033	-5.477	-0.255
Camp 4	-3.174	0.015	-5.688	-0.661
Camp 5	-2.031	0.102	-4.489	0.427
Between*age	6.171	0.001	2.585	9.757
AIC	149.051			

Closeness:

	Interaction model				Non-interaction model			
	B	P	CI	CI	B	P	CI	CI
(Intercept)	1.611	0.207	-0.941	4.163	2.748	0.039	0.153	5.342
Closeness	1.674	0.007	0.493	2.854	1.353	0.034	0.108	2.597
Age	0.671	0.271	-0.553	1.895	-	-	-	-
Camp 1	-0.808	0.593	-3.865	2.248	-1.885	0.236	-5.066	1.296
Camp 2	-2.178	0.129	-5.031	0.676	-3.434	0.022	-6.340	-0.528
Camp 3	-1.308	0.374	-4.269	1.654	-2.410	0.120	-5.479	0.660
Camp 4	-1.763	0.205	-4.547	1.020	-2.834	0.053	-5.703	0.036
Camp 5	-0.757	0.582	-3.541	2.026	-1.925	0.178	-4.776	0.926
Close*age	3.613	0.011	0.885	6.340	-	-	-	-
AIC	156.140				160.723			

Closeness and degree:

	Closeness and degree model			
	B	P	CI	CI
(Intercept)	1.570	0.220	-0.994	4.133
Closeness	1.548	0.015	0.330	2.765
Age	0.566	0.362	-0.685	1.816
Degree	-0.587	0.362	-1.884	0.710
Camp 1	-0.636	0.677	-3.728	2.456
Camp 2	-2.163	0.133	-5.028	0.701
Camp 3	-1.351	0.360	-4.326	1.625
Camp 4	-1.663	0.234	-4.467	1.140
Camp 5	-0.716	0.604	-3.512	2.080
Between*age	3.018	0.051	-0.020	6.055
AIC	156.992			

BaYaka

EC:

	Interaction model				Non-interaction model			
	B	SE	CI	CI	B	P	CI	CI
(Intercept)	-0.435	0.199	-1.110	0.241	-0.441	0.171	-1.083	0.201
EC	0.011	0.984	-1.033	1.055	-0.124	0.764	-0.956	0.709
Age	0.313	0.478	-0.574	1.200	-	-	-	-
Camp 1	0.475	0.291	-0.426	1.376	0.448	0.303	-0.424	1.321
Camp 2	0.549	0.449	-0.913	2.012	0.444	0.523	-0.954	1.841
EC*age	-0.585	0.501	-2.334	1.164	-	-	-	-
AIC	127.950				125.176			

Closeness:

	Interaction model				Non-interaction model			
	B	SE	CI	CI	B	P	CI	CI
(Intercept)	-0.371	0.232	-0.992	0.250	-0.361	0.221	-0.949	0.228
Closeness	0.940	0.025	0.128	1.752	0.962	0.015	0.198	1.725
Age	0.054	0.896	-0.779	0.887	-	-	-	-
Camp 1	0.310	0.467	-0.547	1.167	0.319	0.423	-0.480	1.117
Camp 2	0.306	0.653	-1.069	1.680	0.282	0.657	-0.998	1.562
Close*age	0.270	0.750	-1.442	1.982	-	-	-	-
AIC	127.950				118.562			

Closeness and degree:

	Closeness and degree model			
	B	P	CI	CI
(Intercept)	-0.374	0.226	-0.991	0.243
Closeness	0.930	0.034	0.077	1.783
Age	-0.077	0.858	-0.945	0.791
Degree	0.339	0.419	-0.506	1.184
Camp 1	0.314	0.639	-1.036	1.664
Camp 2	-0.374	0.226	-0.991	0.243
AIC	120.525			

Betweenness:

	Interaction model				Non-interaction model			
	B	SE	CI	CI	B	P	CI	CI
(Intercept)	-0.466	0.138	-1.089	0.158	-0.451	0.132	-1.045	0.143
Betweenness	0.856	0.040	0.042	1.670	0.872	0.029	0.098	1.646
Age	0.137	0.741	-0.697	0.970	-	-	-	-
Camp 1	0.426	0.330	-0.451	1.302	0.427	0.288	-0.378	1.231
Camp 2	0.661	0.340	-0.731	2.053	0.630	0.332	-0.673	1.932
Between*age	0.260	0.806	-1.882	2.402	-	-	-	-
AIC	123.599				119.817			

Betweenness and degree:

	Interaction model			
	B	P	CI	CI
(Intercept)	-0.463	0.136	-1.079	0.153
Closeness	0.834	0.063	-0.048	1.717
Age	-0.085	0.850	-0.985	0.816
Degree	0.446	0.289	-0.397	1.289
Camp 1	0.651	0.331	-0.692	1.994
Camp 2	-0.463	0.136	-1.079	0.153
AIC	121.774			

Strength:

	Interaction model				Non-interaction model			
	B	SE	CI	CI	B	P	CI	CI
(Intercept)	-0.531	0.120	-1.209	0.146	-0.450	0.155	-1.080	0.180
Strength	-0.515	0.276	-1.463	0.433	-0.395	0.331	-1.209	0.419
Age	0.335	0.436	-0.530	1.199	-	-	-	-
Camp 1	0.586	0.207	-0.341	1.513	0.469	0.272	-0.384	1.321
Camp 2	0.590	0.412	-0.859	2.039	0.437	0.522	-0.936	1.811
Strength*age	0.319	0.678	-1.234	1.872	-	-	-	-
AIC	127.273				124.203			

Degree:

	Interaction model				Non-interaction model			
	B	SE	CI	CI	B	P	CI	CI
(Intercept)	-0.559	0.093	-1.217	0.099	-0.517	0.109	-1.154	0.121
Degree	-0.423	0.324	-1.283	0.438	-0.470	0.260	-1.304	0.364
Age	0.251	0.559	-0.614	1.116	-	-	-	-
Camp 1	0.694	0.137	-0.232	1.619	0.564	0.195	-0.302	1.430
Camp 2	0.723	0.319	-0.733	2.180	0.622	0.371	-0.773	2.017
Degree*age	0.659	0.436	-1.046	2.365	-	-	-	-
AIC	126.752				123.832			

Sickness models

Closeness:

	Non-Interaction, non-control model				Dependents control and interaction				Non-interaction with dependents control			
	B	P	CI	CI	B	P	CI	CI	B	P	CI	CI
(Intercept)	0.59	0.29	-0.55	1.73	0.29	0.58	-0.78	1.36	0.21	0.67	-0.80	1.22
Closeness	0.42	0.01	0.10	0.74	0.25	0.15	-0.09	0.59	0.29	0.05	0.01	0.58
Wealth	0.22	0.37	-0.27	0.71	0.00	1.00	-0.45	0.45	0.01	0.95	-0.43	0.46
Age	0.01	0.08	0.00	0.03	0.01	0.08	0.00	0.02	0.01	0.08	0.00	0.02
Mobility (1=no)	-0.51	0.15	-1.21	0.19	-0.42	0.19	-1.06	0.22	-0.38	0.21	-0.99	0.23
Settled (1 = yes)	0.59	0.28	-0.52	1.69	0.09	0.87	-1.00	1.17	0.19	0.69	-0.79	1.18
Camp 1	0.09	0.89	-1.20	1.38	-1.77	0.27	-0.55	1.88	0.58	0.31	-0.58	1.74
Camp 2	-0.53	0.45	-1.96	0.90	-3.72	0.67	-1.11	1.69	0.18	0.78	-1.14	1.50
Camp 3	-0.19	0.57	-0.89	0.51	-2.73	0.98	-0.65	0.66	0.05	0.88	-0.58	0.67
Camp 4	-0.51	0.30	-1.51	0.48	-3.11	0.99	-0.96	0.95	-0.07	0.87	-0.98	0.84
Dependents	-	-	-	-	0.20	0.01	0.06	0.33	0.20	0.00	0.07	0.33
Close*age	-	-	-	-	0.00	0.59	-0.01	0.02	-	-	-	-
AIC	74.92				66.53				64.99			

Betweenness:

	Non-Interaction, non-control model				Dependents control and interaction				Non-interaction with dependents control			
	B	P	CI	CI	B	P	CI	CI	B	P	CI	CI
(Intercept)	1.14	0.00	0.50	1.77	0.85	0.01	0.24	1.47	0.78	0.01	0.18	1.39
Between	0.50	0.04	0.03	0.98	0.49	0.09	-0.09	1.07	0.28	0.20	-0.16	0.72
Wealth	0.67	0.24	-0.49	1.83	0.62	0.03	0.08	1.16	0.22	0.67	-0.84	1.27
Age	0.45	0.26	-0.35	1.25	0.26	0.62	-0.79	1.31	0.10	0.79	-0.64	0.83
Mobility (1=no)	0.54	0.03	0.07	1.01	0.07	0.85	-0.67	0.80	0.43	0.04	0.01	0.84
Settled (1 = yes)	-0.24	0.48	-0.91	0.44	0.56	0.06	-0.02	1.14	0.73	0.01	0.24	1.23
Camp 1	0.22	0.74	-1.12	1.55	-0.26	0.38	-0.87	0.35	-0.17	0.55	-0.76	0.42
Camp 2	-0.82	0.26	-2.28	0.65	0.67	0.26	-0.53	1.88	0.70	0.24	-0.51	1.91
Camp 3	-0.19	0.59	-0.92	0.54	0.01	0.98	-1.37	1.40	0.00	1.00	-1.38	1.39
Camp 4	-0.61	0.24	-1.66	0.43	-0.05	0.88	-0.73	0.63	0.05	0.87	-0.60	0.71
Dependents	-	-	-	-	-0.22	0.65	-1.22	0.77	-0.10	0.83	-1.08	0.87
Between*age	-	-	-	-	0.74	0.26	-0.58	2.05	-	-	-	-
AIC	77.95				68.55				68.54			

EC:

	Non-Interaction, non-control model				Dependents control and interaction				Non-interaction with dependents control			
	B	P	CI	CI	B	P	CI	CI	B	P	CI	CI
(Intercept)	1.13	0.00	0.44	1.81	0.71	0.03	0.09	1.33	0.73	0.02	0.11	1.35
EC	-0.25	0.36	-0.81	0.30	-0.01	0.96	-0.50	0.47	-0.02	0.95	-0.50	0.47
Wealth	0.43	0.09	-0.07	0.92	0.44	0.05	0.00	0.89	0.10	0.85	-1.00	1.19
Age	0.63	0.31	-0.62	1.88	0.01	0.98	-1.10	1.12	0.11	0.77	-0.65	0.87
Mobility (1=no)	0.54	0.21	-0.32	1.39	0.17	0.66	-0.60	0.93	0.36	0.09	-0.06	0.78
Settled (1 = yes)	-0.27	0.48	-1.07	0.52	0.83	0.00	0.32	1.33	0.84	0.00	0.33	1.34
Camp 1	0.21	0.77	-1.24	1.65	-0.09	0.79	-0.76	0.59	-0.12	0.72	-0.79	0.56
Camp 2	-0.83	0.29	-2.41	0.75	0.87	0.17	-0.40	2.14	0.81	0.20	-0.46	2.08
Camp 3	-0.20	0.60	-0.99	0.58	0.08	0.91	-1.35	1.51	0.07	0.92	-1.36	1.50
Camp 4	-0.47	0.39	-1.59	0.64	0.03	0.92	-0.65	0.72	0.08	0.82	-0.60	0.76
Dependents	-	-	-	-	0.10	0.84	-0.89	1.09	0.04	0.93	-0.94	1.03
EC*age	-	-	-	-	-0.45	0.29	-1.31	0.41	-	-	-	-
AIC	82.86				71.22				70.95			

Strength:

	Non-Interaction, non-control model				Dependents control and interaction				Non-interaction with dependents control			
	B	P	CI	CI	B	P	CI	CI	B	P	CI	CI
(Intercept)	1.15	0.00	0.45	1.84	0.66	0.04	0.04	1.29	0.67	0.04	0.05	1.30
Strength	-0.17	0.60	-0.81	0.47	0.13	0.66	-0.47	0.72	0.22	0.43	-0.35	0.79
Wealth	0.42	0.11	-0.09	0.93	0.46	0.04	0.02	0.90	-0.06	0.91	-1.19	1.06
Age	0.64	0.32	-0.66	1.93	-0.14	0.81	-1.27	0.99	0.10	0.78	-0.65	0.85
Mobility (1=no)	0.56	0.19	-0.30	1.42	0.16	0.66	-0.59	0.92	0.39	0.07	-0.03	0.81
Settled (1 = yes)	-0.26	0.56	-1.15	0.64	0.87	0.00	0.34	1.39	0.92	0.00	0.40	1.44
Camp 1	0.20	0.78	-1.30	1.70	0.09	0.80	-0.66	0.84	0.08	0.84	-0.68	0.83
Camp 2	-0.89	0.26	-2.48	0.70	0.99	0.13	-0.32	2.29	1.00	0.13	-0.30	2.31
Camp 3	-0.22	0.58	-1.01	0.57	0.11	0.87	-1.30	1.53	0.11	0.87	-1.31	1.53
Camp 4	-0.49	0.38	-1.62	0.64	0.03	0.93	-0.65	0.71	0.10	0.76	-0.57	0.77
Dependents	-	-	-	-	0.15	0.75	-0.83	1.14	0.12	0.80	-0.86	1.11
Strength*age	-	-	-	-	-0.60	0.26	-1.68	0.48	-	-	-	-
AIC	83.64				70.06				70.01			

Degree:

	Non-Interaction, non-control model				Dependents control and interaction				Non-interaction with dependents control			
	B	P	CI	CI	B	P	CI	CI	B	P	CI	CI
(Intercept)	1.13	0.00	0.42	1.84	0.64	0.04	0.02	1.25	0.75	0.02	0.13	1.37
Degree	-0.02	0.94	-0.55	0.50	0.03	0.89	-0.40	0.46	0.11	0.62	-0.33	0.54
Wealth	0.44	0.09	-0.07	0.95	0.45	0.03	0.04	0.86	0.12	0.82	-0.95	1.19
Age	0.54	0.39	-0.73	1.82	-0.19	0.72	-1.28	0.91	0.12	0.74	-0.64	0.88
Mobility (1=no)	0.58	0.18	-0.28	1.45	0.04	0.92	-0.70	0.77	0.37	0.08	-0.05	0.78
Settled (1 = yes)	-0.13	0.73	-0.92	0.65	0.82	0.00	0.35	1.29	0.86	0.00	0.37	1.35
Camp 1	0.32	0.67	-1.17	1.80	-0.10	0.73	-0.73	0.52	-0.05	0.88	-0.69	0.60
Camp 2	-0.92	0.26	-2.54	0.71	0.98	0.11	-0.25	2.20	0.77	0.21	-0.47	2.02
Camp 3	-0.23	0.57	-1.03	0.58	0.34	0.62	-1.09	1.78	0.02	0.97	-1.41	1.46
Camp 4	-0.46	0.42	-1.61	0.69	-0.05	0.88	-0.74	0.64	0.11	0.75	-0.58	0.79
Dependents	-	-	-	-	0.17	0.72	-0.80	1.14	0.01	0.98	-0.97	1.00
Degree*age	-	-	-	-	-0.70	0.10	-1.54	0.14	-	-	-	-
AIC	84.04				68.34				70.59			
